# FAK Regulates VEGFR2 Expression and Promotes Angiogenesis in Triple-Negative Breast Cancer

**DOI:** 10.3390/biomedicines9121789

**Published:** 2021-11-29

**Authors:** Jun-Ping Shiau, Cheng-Che Wu, Shu-Jyuan Chang, Mei-Ren Pan, Wangta Liu, Fu Ou-Yang, Fang-Ming Chen, Ming-Feng Hou, Shen-Liang Shih, Chi-Wen Luo

**Affiliations:** 1 Division of Breast Oncology and Surgery, Department of Surgery, Kaohsiung Medical University Hospital, Kaohsiung 807, Taiwan; gp5066@gmail.com (J.-P.S.); asabolu0122@yahoo.com.tw (C.-C.W.); kmufrank@gmail.com (F.O.-Y.); fmc5464@gmail.com (F.-M.C.); mifeho@kmu.edu.tw (M.-F.H.); slshih1@gmail.com (S.-L.S.); 2 Department of Surgery, Kaohsiung Municipal Siaogang Hospital, Kaohsiung 812, Taiwan; 3 Department of Pathology, Kaohsiung Medical University Hospital, Kaohsiung 807, Taiwan; binbin4728@gmail.com; 4 Department of Pathology, College of Medicine, Kaohsiung Medical University, Kaohsiung 807, Taiwan; 5 Graduate Institute of Clinical Medicine, Kaohsiung Medical University, Kaohsiung 807, Taiwan; pan.meiren0324@gmail.com; 6 Drug Development and Value Creation Research Center, Kaohsiung Medical University, Kaohsiung 807, Taiwan; 7 Department of Biotechnology, Kaohsiung Medical University, Kaohsiung 807, Taiwan; liuwangta@kmu.edu.tw; 8 Center for Cancer Research, Kaohsiung Medical University, Kaohsiung 807, Taiwan; 9 Department of Medical Research, Kaohsiung Medical University Hospital, Kaohsiung 807, Taiwan; 10 Department of Biomedical Science and Environmental Biology, College of Life Science, Kaohsiung Medical University, Kaohsiung 807, Taiwan; 11 Center for Liquid Biopsy and Cohort Research, Kaohsiung Medical University, Kaohsiung 807, Taiwan

**Keywords:** focal adhesion kinase (FAK), vascular endothelial growth factor 2 (VEGFR2), triple-negative breast cancer (TNBC), angiogenesis

## Abstract

Triple-negative breast cancer (TNBC) remains a significant clinical challenge because of its high vascularity and metastatic and recurrent rates. Tumor angiogenesis is considered an important mediator in the regulation of tumor cell survival and metastasis in TNBC. Angiogenesis is induced by the binding of vascular endothelial growth factor to vascular endothelial growth factor receptor 2 (VEGFR2). Focal adhesion kinase (FAK) plays an important role in regulating various cell functions in normal and cancer cells. Previous studies have focused on investigating the function of endothelial FAK in tumor cell angiogenesis. However, the association between tumor FAK and VEGFR2 in tumor angiogenesis and the possible mechanisms of this remain unclear. In this study, we used a public database and human specimens to examine the association between FAK and VEGFR2. At the same time, we verified the association between FAK and VEGFR2 through several experimental methods, such as quantitative real-time polymerase chain reaction, Western blotting, and next-generation sequencing. In addition, we used the endothelial cell model, zebrafish, and xenograft animal models to investigate the role of FAK in TNBC angiogenesis. We found that FAK and VEGFR2 were positively correlated in patients with TNBC. VEGFR2 and several other angiogenesis-related genes were regulated by FAK. In addition, FAK regulated VEGFR2 and VEGF protein expression in TNBC cells. Functional assays showed that FAK knockdown inhibited endothelial tube formation and zebrafish angiogenesis. An animal model showed that FAK inhibitors could suppress tumor growth and tumor vascular formation. FAK promotes angiogenesis in TNBC cells by regulating VEGFR2 expression. Therefore, targeting FAK could be another antiangiogenic strategy for TNBC treatment.

## 1. Introduction

Breast cancer is a heterogeneous disease and the most common cancer type worldwide [1]. Although diagnostic methods and therapeutic strategies have made significant progress in breast cancer, it still represents the most common leading cause of cancer-related deaths among women worldwide [2]. Among all breast cancer subtypes, triple-negative breast cancer (TNBC) is the most aggressive subtype with a high probability of angiogenesis, metastasis, and drug resistance and a lack of specific targets and targeted therapies [3]. Previous studies have indicated that angiogenesis is essential for the dissemination and establishment of tumor metastases, and these phenomena are regulated by the interactions between tumor cells and tumor microenvironment (TME) [4,5]. Therefore, understanding the TME and its regulators will be useful for developing new targeted therapies to decrease tumor progression and metastasis in patients with TNBC.

Angiogenesis plays an important role in tumor formation, progression, and metastasis [6]. Previous studies have indicated that vascular endothelial growth factors (VEGFs) and their receptors (VEGFRs) are considered the most critical pro-angiogenic factors that regulate tumor angiogenesis [7,8,9,10]. Cancer cells secrete VEGFA and activate VEGFR2 on their surface to form an autocrine loop, which enables cancer cells to promote their own growth and survival [11]. Therefore, the VEGF/VEGFR2 system is an important target for developing antiangiogenic therapies [12,13]. Prior studies have indicated that TNBC has higher intratumoral VEGF levels and VEGFR2 expression and shorter survival times compared with non-TNBC [14,15]. Hence, angiogenesis is an important process in the regulation of TNBC progression and metastasis. Most previous studies have focused on the role of angiogenesis in endothelial cells, but not in tumor cells; therefore, it is necessary to investigate the role of angiogenesis in tumor cells.

Focal adhesion kinase (FAK), a non-receptor protein tyrosine kinase, is mainly regulated by integrin signaling [16]. FAK overexpression has been observed in various types of cancer cells and vascular cells surrounding solid tumors [7]. FAK regulates various functions, such as survival, proliferation, angiogenesis, metastasis, TME regulation, and chemoresistance in normal and cancer cells [16,17,18,19,20]. In addition, FAK also plays a role in regulating the drug response of immunotherapies [21,22], suggesting that FAK could be a target for cancer treatment. Several FAK inhibitors have been developed and tested in clinical trials [23]. Previous studies have indicated that FAK plays a role in regulating tumor angiogenesis. They suggested that FAK and several other proteins (such as protein kinase B and mammalian target of rapamycin) could be phosphorylated by VEGFR2 after activation by VEGF [24,25,26], stimulating tumor cell proliferation, survival, migration, metastasis, and angiogenesis. Although most studies suggested that FAK could be downstream of VEGFR2, however, several recent studies indicated that the nuclear FAK and its kinase activity could transcriptionally regulate VEGFR2 expression and that FAK-Y397F reduced VEGFR2 expression in endothelial cells [27,28]. Therefore, FAK is not only downstream of VEGFR2, but it also plays a role in regulating VEGFR2 expression. However, these studies have focused on the role of FAK in endothelial cells. Whether FAK also plays a role in regulating angiogenic pathways within tumor cells remains unclear. Based on this background, we aimed to investigate whether FAK plays a role in regulating tumor angiogenesis in TNBC.

## 2. Materials and Methods

### 2.1. Cell Lines and Cell Culture

The human TNBC cancer cell lines MDA-MB-231 and MDA-MB-468 (ATCC, Manassas, VA, USA) were used in this study. In addition, human umbilical vein endothelial cells (HUVECs) (BCRC, Hsinchu, Taiwan) were also used in this study. MDA-MB-231 and MDA-MB-468 cells were maintained in Dulbecco’s Modified Eagle’s Medium (Gibco; Thermo Fisher Scientific, Inc., Waltham, MA, USA) supplemented with 10% fetal bovine serum (Gibco; Thermo Fisher Scientific, Inc., Waltham, MA, USA), 100 U penicillin, and 100 mg/mL streptomycin (Gibco). HUVECs were maintained in M199 Medium (Gibco) with growth factors according to the manufacturer’s protocol. All cells were incubated in a humidified atmosphere at 37 °C with 5% carbon dioxide.

### 2.2. Antibodies and Reagents

Antibodies against FAK (#3285), p-FAK (#3283), VEGFR2 (#2479S), CD31(#77699), and GAPDH (#2118) were purchased from Cell Signaling Technology (Beverly, MA, USA). An antibody against VEGF (MA5-13182) was purchased from Thermo Fisher Scientific, Inc. Moreover, mouse (GTX213111), rabbit (GTX213110), IgG (HRP), and p-FAK (GTX129840) antibodies were purchased from GeneTex Inc. (Hsinchu City, Taiwan, R.O.C.) and used as secondary antibodies. PF-562271, a FAK inhibitor, was purchased from Selleckchem (Houston, TX, USA).

### 2.3. Western Blotting

Protein extraction and immunoblotting were performed as previously described [20,29]. Briefly, cells were lysed in a protein extraction buffer (Thermo Fisher Scientific, Inc.). Next, the proteins were collected, loaded on a sodium dodecyl sulfate–polyacrylamide gel, and transferred to nitrocellulose membranes. Immunoblotting was performed using primary and secondary antibodies. Signals were detected using a chemiluminescence assay and captured using the ChemiDoc XRS+ system (Bio-Rad Laboratories, Inc., Hercules, CA, USA).

### 2.4. Short Interfering RNA and Plasmid of FAK Transfections

Short interfering RNA (siRNA) for human FAK (PTK2, M-003164-02-0005) and negative control (CN-001000-01-05) was purchased from Dharmacon (Dharmacon Life Technologies, Cologne, Germany). Plasmid constitutive expressing a full-length wild-type FAK protein was obtained from Genecopoeia Inc. Cells were transfected with the suitable amount of appropriate non-targeting and specific siRNAs or expression construct and control empty vector by using Lipofectamine 2000 (Thermo Fisher Scientific, Inc.), following the manufacturer’s protocol.

### 2.5. FAK shRNA Clone Transfection

shRNA clones were obtained from the National RNAi Core Facility Platform from Academia Sinica, Taiwan. To knock down the expression of FAK in MDA-MB-231 cells, shRNA against human FAK (shFAK) (sequence: #1: GATGTTGGTTTAAAGCGATTT, #2: CCCAGGAGAGAATGAAGCAAA, and #3: CCGATTGGAAACCAACATATA) and shRNA against luciferase (sequence: GCGGTTGCCAAGAGGTTCCAT) were used in this study. Transfected cells were selected using puromycin, and the efficiency of FAK silencing was evaluated using real-time reverse transcription–polymerase chain reaction.

### 2.6. RNA Extraction, Genome-Wide Transcriptome Characterization, and Expression Profiling by RNA Sequencing

Next-generation sequencing (NGS) is a technology that determines the sequence of DNA or RNA to study genetic variation associated with diseases or other biological phenomena. In this study, we applied the NGS technology to investigate the differences in gene expressions between parental (shLuci) and stable knockdown FAK (shFAK) MDA-MB-231 cells. In brief, the methods of extraction of total RNA from shLuci and shFAK MDA-MB-231 cells and the evaluation of the extracted RNA quality were similar to our previous study [30]. Subsequent analyses were performed by Genewiz Company (AllBio Science, Inc., Taipei, Taiwan). mRNA from shLuci and shFAK cells was considered to be differentially expressed when a fold-change > 2.0 was observed and fragments per kilobase of transcript per million (FPKM) > 0.2 for mRNA was observed. Using FPKM > 0.2 as the threshold for RNA-seq decreased the number of false-positive results/negative detection and increased the confidence in the measured expression level.

### 2.7. Quantitative Real-Time Polymerase Chain Reaction

Total RNA extraction and quantitative polymerase chain reaction (q-PCR) protocols were performed as described in our previous study [30]. Synthesized cDNA was used as a template for PCR amplification with primers for human FAK (primers forward, 5′-AGATCCTGTCTCCAGTCTAC-3′, and reverse, 5′-AATGGTTTGCACTTGAGTGA-3), VEGFR2 (primers forward, VEGFR2-F: 5′-CGGACAGTGGTATGGTTCTTGC-3, and reverse, 5′-GTGGTGTCTGTGTCATCGGAGTG-3), and GAPDH (primers forward, 5′-AAGGCTGGGGCTCATTTGC-3′, and reverse, 5′-GCTGATGATCTTGAGGCT-3′). Quantitative real-time PCR was performed in a 20-μL reaction volume using the standard protocols provided by the Roche LightCycler 480 II system (Basel, Switzerland). FAK and VEGFR2 gene expressions were determined as follows: ΔCT = CT (target gene) − CT (GAPDH) and ΔΔCT = ΔCT (experimental group) − ΔCT (control group).

### 2.8. Preparation of Conditioned Medium and Matrigel Tube Formation Experiment

The preparation methods of conditioned medium (CM) were similar to those reported in other studies [31]. In brief, shLuci and shFAK MDA-MB-231 cells were seeded in 6-well plates for 24 h, washed twice with phosphate-buffered saline to remove serum components, replaced with a serum-free medium, and incubated for another 48 h. The supernatants were collected, centrifuged, filtered, and used in TCM for further study. For tube formation experiments, HUVECs at a density of 3 × 10^4^ cells per well were grown on top of Matrigel (BD Biosciences, Billerica, MA, USA) and cultured in CM. After incubation for 2–4 h at 37 °C, the tube structures were photographed by microscopy.

### 2.9. Human Specimens

Breast cancer tissue microarray (CBA4, SuperBioChips Laboratories, Seoul, Korea) was used in this study. The details on clinicopathological and survival information of patients were provided on the manufacturer’s websites (https://www.tissue-array.com/main.html, accessed on 10 January 2021). Several clinical biomarker expressions such as estrogen receptor (ER), progesterone receptor (PR), human epidermal growth factor receptor 2 (HER2), and Ki-67 were provided by the manufacturers on their websites. In total, 15 cases of TNBC and 25 cases of non-TNBC were collected in this tissue microarray.

### 2.10. Ethics Statement

Animal experimental procedures were approved by the Institute of Animal Care and Use Committee at Kaohsiung Medical University and performed in accordance with the Guide for the Care and Use of Laboratory Animals. Animals were housed in an Association for Assessment and Accreditation of Laboratory Animal Care International approved animal facility at Kaohsiung Medical University.

### 2.11. Animal Grouping

MDA-MB-231 cells (2 × 10^6^; 1:1 mixed with Matrigel) were injected subcutaneously into the fat pad position of 8-week-old female nude mice (National Laboratory Animal Center, Taipei City, Taiwan). Tumor volume was measured using a caliper and calculated according to the following formula: tumor volume = (length × width^2^)/2. When tumor volume reached approximately 100 mm^3^, the tumor-bearing mice were randomized and treated with PF-562271 (50 mg/kg) or dimethylsulfoxide intraperitoneally as a twice-daily treatment. Tumor specimens were subjected to immunohistochemical (IHC) staining.

### 2.12. Zebrafish Assay

Zebrafish (strain fli1: enhanced green fluorescent protein (EGFP) from the Zebrafish Core Facility, Center for laboratory animals, Kaohsiung Medical University, Kaohsiung, Taiwan), which have an eGFP under the fli1 promoter expressed specifically in endothelial cells allowing the visualization of both blood and lymphatic vascular systems, were used for our study. The zebrafish angiogenesis assay was similar to other reported studies [32]. In brief, shLuci and shFAK MDA-MB-231 cancer cells were labeled with PKH26 Red Fluorescent Cell Linker Kit (Merck KGaA, Darmstadt, Germany) and implanted into the perivitelline cavity of 2-day-old zebrafish embryos through microinjection. After confirmation of the localized PKH26-labeled cell mass at the injection site, the zebrafish embryos were transferred to fresh water and maintained at 32.5 °C; subsequently, vascular sprouts were observed using a Nikon Eclipse Ti-S 217 microscope (Tokyo, Japan).

### 2.13. Immunohistochemical Staining and Scoring

Paraffin-embedded tissue sections were de-paraffinized; rehydrated; retrieved; treated with hydrogen peroxide; incubated with primary antibodies overnight; and then incubated with the Envision systems (Dako, Denmark). Finally, the sections were counterstained with hematoxylin and analyzed under a microscope. FAK and VEGFR2 expressions were defined as cytoplasmic staining in tumor cells and scored based on the product of signal intensity and the proportion of positive cells [21].

### 2.14. Statistical Analysis

Statistical differences between each group were compared and calculated using a two-tailed Student’s *t*-test. *p* < 0.05 was considered statistically significant. All statistical analyses were performed using the Statistical Package for the Social Sciences version 19.0 software (IBM Corp., Armonk, NY, USA).

## 3. Results

### 3.1. FAK Expression Is Positively Correlated with VEGFR2 Expression in Patients with TNBC

**Figure 1:**
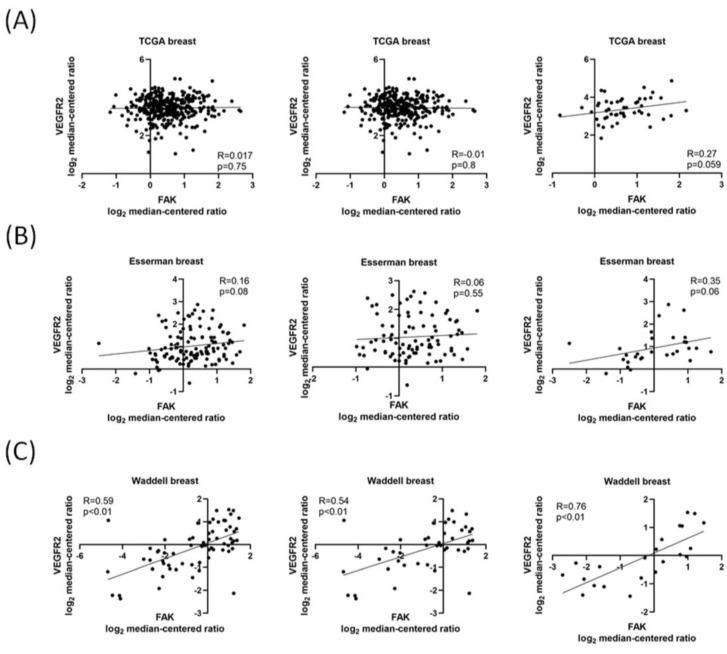
FAK gene expression is positively correlated with VEGFR2 expression in patients with TNBC. Data sets were categorized as “Total breast cancer”, “non-TNBC” (if tumors were positive for either ER, PR, or ERBB2), and “TNBC” (if negative for ER, PR, and ERBB2). These data were obtained from the Oncomine database (https://www.oncomine.com, accessed on 25 July 2021). FAK and VEGFR2 expression were analyzed from (**A**) TCGA, (**B**) Esserman breast, and (**C**) Waddell breast data sets.

FAK has been considered a mediator of tumor angiogenesis in vascular cells surrounding solid tumors [16], and nuclear FAK activity plays a role in endothelial cells during tumor angiogenesis [27]. In addition, VEGFR2 has been indicated as a key factor of angiogenesis in many previous studies. Therefore, we first used Oncomine, a public database of different expressed genes, to explore the association between FAK and VEGFR2 in patients with TNBC. By examining several gene expression data sets, we found that there was a positive correlation between FAK and VEGFR2, especially in patients with TNBC (Figure 1A–C).

**Figure 2:**
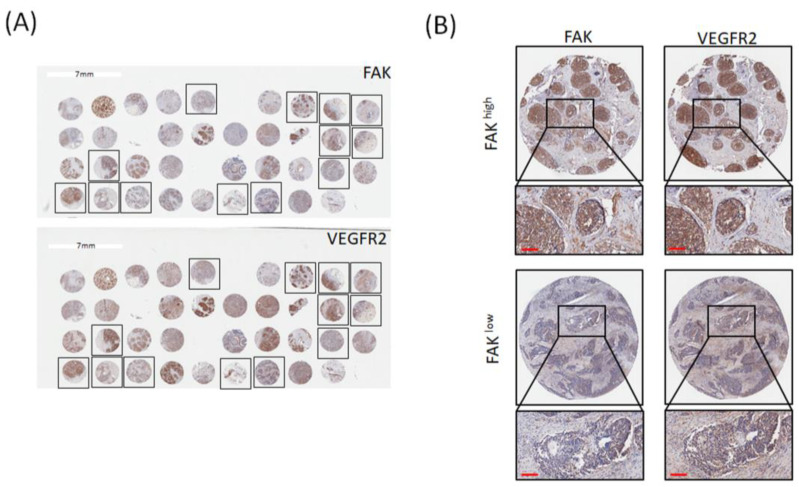
FAK protein expression is correlated with VEGFR2 expression. (**A**) Representative IHC staining results for expression of FAK and VEGFR2 in breast cancer tissue microarrays. (**B**) Immunoreactivity of FAK and VEGFR2 was classified as high expression (**upper**) and low expression (**lower**) based on staining observed for the cell cytoplasm and membrane. (Scale bar: 100 μm).

To further elucidate that FAK was positively correlated with VEGFR2 in patients with TNBC, we used the tissue microarrays (TMA) to investigate the relationships between FAK and VEGFR2. Figure 2A represents the IHC staining of FAK and VEGFR2 in all breast cancer tissues, and the frames are TNBC subtypes. Figure 2B shows high expression and low expression of FAK and VEGFR2 in the same patients. We found that there was a positive correlation between FAK and VEGFR2 protein expressions in TNBC subtypes, which is similar with the analytic results from the public database (Table 1), suggested that FAK gene and protein expressions are associated with VEGFR2 gene and protein expressions. Therefore, FAK was positively correlated with VEGFR2 expression in patients with TNBC.

### 3.2. FAK Plays a Role in Regulating VEGFR2 Gene Expression in TNBC Cells

**Figure 3:**
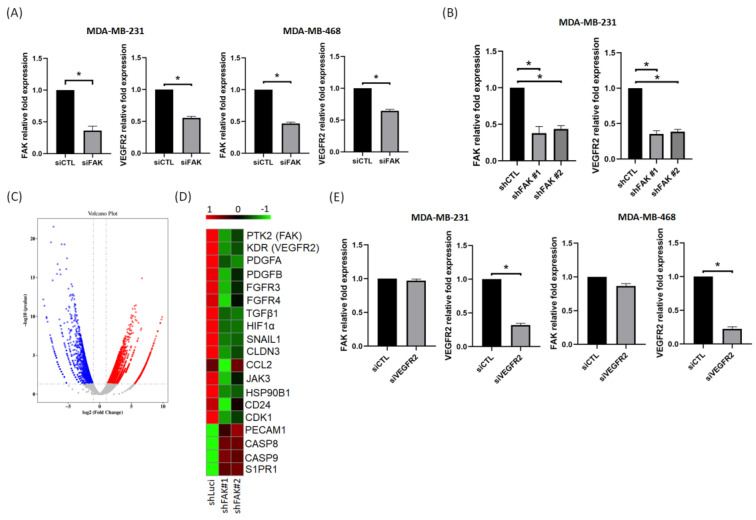
VEGFR2 is regulated through gene expression of FAK in TNBC cells. (**A**) Knockdown of FAK decreased VEGFR2 gene expression in MDA-MB-231 and MDA-MB-468 cells. (**B**) VEGFR2 gene expression decreased in two stable knockdown FAK clones. (**C**) The different gene expressions in shLuci and two shFAK MDA-MB-231 cells from RNA sequencing displayed as a volcano plot. Red marks represent upregulated genes and blue marks represent downregulated genes. (**D**) Heatmap shows that several angiogenic related gene expressions, such as PTK2, KDR, TGF-β1, HIF-1α, etc., were reduced significantly in shFAK TNBC cells. (**E**) Knockdown of VEGFR2 expression could not affect FAK gene expression in MDA-MB-231 and MDA-MB-468 cells. Data from three independent experiments were used for statistical analysis and *: *p* < 0.05.

From the analysis of several data sets, we found that VEGFR2 was positively correlated with FAK. In addition, IHC staining also showed that the FAK protein expression was also positively correlated with VEGFR2. Previous studies have indicated that the VEGF–VEGFR2 feed-forward loop in cancer cells plays a dominant role in promoting tumor angiogenesis and metastasis [33]. Therefore, we investigated whether this VEGF–VEGFR2 mediated feed-forward loop could be regulated by FAK in TNBC cells. Our results showed that VEGFR2 mRNA expression was downregulated in MDA-MB-231 and MDA-MB-468 cells after a knockdown of FAK by siRNA in both TNBC cells (Figure 3A). Next, we established two stable FAK knockdown MDA-MB-231 clones (shFAK) to compare gene expressions with shLuci MDA-MB-231 cells. Analytic results indicated that the expression of VEGFR2 was reduced in shFAK MDA-MB-231 cells (Figure 3B). In addition, the NGS data showed that there were 5180 significantly differentially expressed genes (2819 upregulated and 2361 downregulated) between shLuci and shFAK MDA-MB-231 cells (Figure 3C). Among these gene expression profiles, VEGFR2 gene expression decreased significantly in shFAK MDA-MB-231 cells, and several angiogenic-related genes were also downregulated in shFAK MDA-MB-231 cells (Figure 3D). However, we found that FAK gene expression did not change significantly in knockdown VEGFR2 cells (Figure 3E), suggesting that FAK could be a mediator of VEGFR2. Therefore, FAK can regulate angiogenesis by mediating the VEGFR2-related pathways in TNBC cells.

### 3.3. FAK Plays Roles in Regulating VEGFR2 Protein Expressions in TNBC Cells

**Figure 4:**
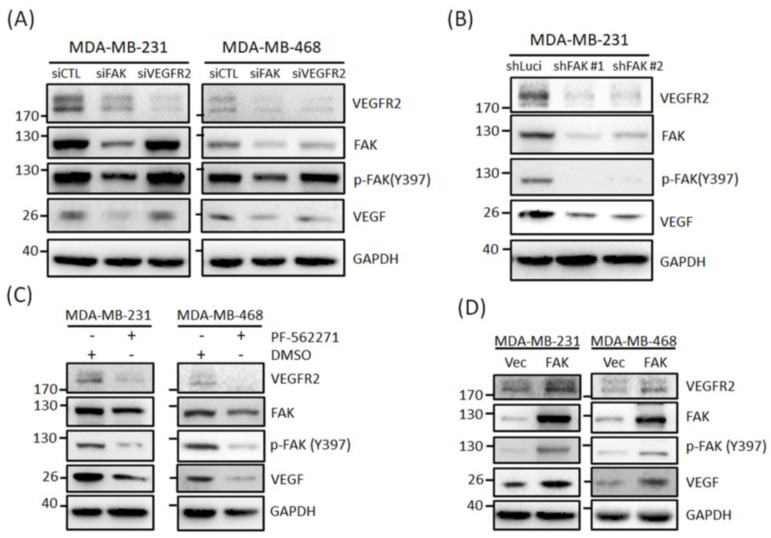
VEGFR2 protein expression is modulated through gene regulation by FAK in TNBC cells. (**A**) Knockdown of FAK decreased protein expressions of VEGFR2, p-FAK, and VEGF but knockdown of VEGFR2 did not affect these protein expressions in MDA-MB-231 and MDA-468 cells. (**B**) Protein expressions of VEGFR2, p-FAK, and VEGF were reduced in two stable clones of knockdown FAK MDA-Mb-231 cells. (**C**) VEGFR2 protein expressions in MDA-MB-231 and MDA-MB-468 cells were significant suppressed through PF-562271 treatment. (**D**) Overexpression of FAK could also increase VEGFR2, p-FAK, and VEGF protein expression in MDA-MB-231 and MDA-MB-468 cells.

We have shown that FAK regulates VEGFR2 expression in TNBC cells. Next, we investigated whether FAK could also affect VEGFR2 protein expression in TNBC cells. Our Western blotting results showed that FAK knockdown suppressed VEGFR2 and VEGF protein expressions in MDA-MB-231 and MDA-MB-468 cells, but the knockdown of VEGFR2 did not affect FAK and VEGF expression (Figure 4A). These VEGFR2 and VEGF inhibitory effects were also observed in shLuci and shFAK MDA-MB-231 cells (Figure 4B). Additionally, we used an FAK inhibitor (PF-562271) to confirm its inhibitory effects. As shown in Figure 4C, PF-562271 significantly suppressed p-FAK(Y397), VEGFR2, and VEGF protein expressions in both TNBC cell lines, suggesting that FAK inhibition could be another way to inhibit angiogenesis in TNBC. We also transfected the FAK plasmid into MDA-MB-231 and MDA-MB-468 cells to confirm that FAK could regulate VEGFR2 expression. Figure 4D shows that the overexpression of FAK in both TNBC cells increased VEGFR2 and VEGF expressions. Therefore, FAK could mediate TNBC cell angiogenesis by regulating VEGFR2 gene and protein expressions.

### 3.4. Suppression of FAK Affects the Tumor Angiogenesis and Growth In Vitro and In Vivo

**Figure 5:**
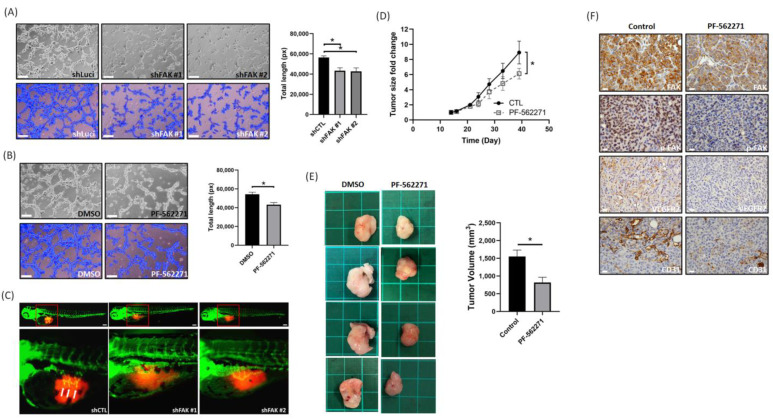
Suppression of FAK affects TNBC cell angiogenesis and tumor growth in vitro and in vivo. (**A**) Representative images of tube formation in HUVECs treated with CM derived from shLuci MDA-MB-231 cells and shFAK MDA-MB-231 cells. Scale bar: 100 μm. (**B**) Representative images of tube formation in HUVECs treated with CM derived from MDA-MB-231 cells and PF-562271 treated MDA-MB-231 cells. Scale bar: 100 μm. (**C**) The sprouts originating from the subintestinal venous plexus in the embryos (the white arrow pointed position) of zebrafish showed that shLuci MDA-MB-231 cells induced tumor angiogenesis, but this was suppressed in shFAK MDA-MB-231 cells. Scale bar: 100 μm. (**D**) PF-562271 suppressed tumor growth in MDA-MB-231 xenograft mice model (*n* = 4 for each group). (**E**) Tumor volume between vehicle and PF-562271 treated groups. Each tumor was from a different mouse. The bar chart represents the quantification of tumor volume from mice (*n* = 4 for each group). Tumor volume in PF-562271 treated group was significantly smaller than in vehicle treated group. (**F**) IHC staining represents FAK, p-FAK(Y397), VEGFR2, and CD31 expressions. The expressions of these proteins were reduced in PF-562271 treated group than vehicle treated group. Scale bar: 20 μm. *: *p* < 0.05.

According to our in vitro experimental results, we found that FAK could regulate the VEGF–VEGFR2 feed-forward loop by regulating VEGFR2 and VEGF expressions in MDA-MB-231 and MDA-MB-468 cells. To confirm these findings, we used the HUVEC tube formation assay, zebrafish, and xenograft models to verify our hypothesis. As shown in Figure 5A, the number of tubes in the shPTK2 group was less than that in the control group. These results indicated that CM from shFAK cells significantly inhibited tube formation ability compared to that of CM from shLuci cells. In addition, we also found that PF-562271-treated CM could also inhibit tube formation (Figure 5B), suggesting that inhibition induces an antiangiogenic effect. A previous study indicated that the sprouts originating from the subintestinal venous plexus in the embryos of zebrafish could be an indicator of angiogenesis [32]. In this study, our results showed that the injection of shFAK MDA-MB-231 cells (shFAK#1 and #2) led to a decrease in ectopic sprouts originating from the subintestinal venous plexus in the embryos of zebrafish compared to the injection of shLuci MDA-MB-231 cells (Figure 5C). In the xenograft model, tumor growth rate and tumor volume were reduced in mice treated with PF-562271 compared to mice treated with vehicle only (Figure 5D,E), suggesting that PF-562271 could suppress tumor growth in TNBC cells. Next, IHC staining showed that VEGFR2 and FAK expressions were lower in mice treated with PF-562271 compared to the control mice (Figure 5F). In addition, CD31 (platelet endothelial cell adhesion molecule-1, PECAM-1) is used primarily to demonstrate the presence of endothelial cells in histological tissue sections and involved in angiogenesis [34]. Therefore, we used CD31 IHC staining to evaluate the degree of tumor angiogenesis. The amount of CD31 expression in mice treated with PF-562271 was also less than that in the control mice (Figure 4F), suggesting that PF-562271 suppresses the formation of tumor vessels. These results suggest that FAK inhibition could inhibit angiogenesis in TNBC cells through regulating the FAK–VEGFR2 related signaling pathway.

## 4. Discussion

Tumor angiogenesis is a process that promotes new blood vessel formation from pre-existing vessels. The sprouting of new blood vessels not only provides nutrients and oxygen to tumor cells but also forms a route to help tumor cell dissemination and establish distal metastasis [35,36]. These phenomena are regulated not only through tumor cells but also through the interactions between tumor cells and their TME. Among the angiogenic factors, VEGF is the strongest mediator in the regulation of angiogenesis. Endothelial cell survival, migration, and proliferation of blood vessels are regulated primarily by VEGF-A binding to VEGFRs. In addition, VEGF secreted from tumor cells also binds to the VEGFRs on their cell surface, enabling tumor cells to promote their own growth and survival [10,11,37,38,39]. Among all VEGFRs, VEGFR2 plays a major role in mediating the VEGF-induced series of downstream signals of angiogenesis [40]. Previous studies have indicated that VEGFR2 is abundantly expressed in endothelial cells and several cancer cells [13,41,42,43,44,45]. Thus, targeting the VEGFR2 signaling pathway to inhibit tumor angiogenesis is considered another antiangiogenic strategy. Overall, the inhibition of tumor angiogenesis could be performed by blocking the VEGF/VEGFR2 signaling pathway.

TNBC is a unique subtype of breast cancer that is associated with an aggressive phenotype, high recurrence, metastasis, and poor prognosis. In addition, the lymphatic vessels and microvascular density in TNBC are significantly higher than in non-TNBC, and several genes involved in angiogenesis are frequently activated in basal-like breast cancer [5,46], suggesting that the inhibition of angiogenesis-related pathways could be a potential strategy for the treatment of TNBC [47]. To date, the only antiangiogenic drug approved by the Food and Drug Administration (FDA) is bevacizumab (Avastin). It is a recombinant humanized monoclonal antibody that blocks angiogenesis by inhibiting VEGFA and it provides new hope for improved survival in patients with TNBC in combination with chemotherapies. However, this treatment cannot fully meet the expectations of patients regarding higher overall survival because of drug resistance. Therefore, scientists have investigated the role of VEGF receptors and developed several VEGFR inhibitors as other choices for antiangiogenic therapies [13]. Although VEGFR2 is also an important target for antitumor and antiangiogenic therapies, several VEGFR2 inhibitors have been approved for clinical use; however, no VEGFR2 inhibitors have been approved for TNBC treatment by FDA. This implies that there should be other genes participating in regulating VEGF/VEGFR2 expression in TNBC. Thus, it is necessary to explore alternative antiangiogenic agents to improve their therapeutic effectiveness.

FAK is considered an oncoprotein in many types of cancers. In addition to its pro-tumor roles, FAK can also affect several malignant characteristics, such as tumor growth, metastasis, and chemoresistance of tumor cells by regulating angiogenesis, vascular permeability, and fibrosis through other cells within the TME [2,16,17,19,21]. The development of novel FAK inhibitors is currently in progress, and several FAK inhibitors have been used in clinical trials [23]. Many of these showed therapeutic effects on cancer treatment, suggesting that this is one of the directions for anti-cancer drug development. To date, many studies have focused on the role of FAK in regulating angiogenesis in endothelial cells, and most of these studies suggested that VEGFR2 activation by VEGF stimulates FAK phosphorylation and this is required for promoting endothelial cell migration. However, a prior study also suggested that intrinsic FAK activity facilitates an angiogenic switch in tumors, and several recent studies also indicated that FAK and its different kinase activity were critical for regulating VEGFR2 transcription and expression [27,28]. In addition, our previous study also found that VEGFR2 seems to positive correlated with FAK in tumor tissues [17]. Therefore, we supposed that FAK could play roles in regulating angiogenesis through mediating VEGFR2 expression in tumor cells.

In this study, we found that VEGFR2 gene and protein expression levels were positively correlated with FAK in patients with TNBC through a public database (Oncomine) and commercial tissue microarray. Our in vitro studies indicated that the inhibition of FAK gene expression suppressed VEGFR2 gene and protein expressions. Conversely, FAK overexpression increased VEGFR2 protein expression. In addition, VEGF protein expression was also reduced in FAK knockdown cells. VEGFR2 and several other angiogenesis-related genes (such as hypoxia-inducible factor 1-alpha (HIF-1α), transforming growth factor beta-1 (TGF-β1), platelet-derived growth factor A (PDGFA), and PDGFB) are also downregulated in FAK knockdown cells from NGS data. FAK overexpression increased VEGFR2 protein expression. These abovementioned data suggest that VEGFR2 expression and protein expression could be regulated by FAK in TNBC cells. Furthermore, our functional assay showed that the conditioned medium from FAK knockdown and FAK inhibitor-treated TNBC cells reduced HUVEC tube formation. Our zebrafish model also showed that subintestinal venous plexus sprouting, an indicator of tumor angiogenesis, was also inhibited in FAK knockdown TNBC cells. In a xenograft mouse model, we found that FAK inhibitors could suppress tumor growth and decrease vessel formation. These results indicate that FAK can modulate VEGFR2 expression and promote angiogenesis in TNBC cells, and FAK inhibitors could be another antiangiogenic agent for TNBC treatment.

PF-562271 is a selective inhibitor of both FAK and proline-rich tyrosine kinase 2 (PYK2) [48]. It is well known that Pyk2 is the only other kinase that is structurally and functionally related to FAK. Previous studies found that compensatory Pyk2 upregulation is often observed when FAK is deleted or inhibited [49]. In our study, we found that, whether causing a knockdown of FAK by siRNA and shRNA or by using PF-562271, these all reduced the p-FAK expression and VEGFR2 expression in TNBC cells and reduced the tube formation of endothelial cells. In addition, our animal model also showed that PF-562271 could also suppress the tumor growth and several protein expressions (such as FAK, p-FAK, and CD31), suggesting that PF-562271 may suppress tumor angiogenesis mainly through the inhibition of p-FAK expression in tumor cells. However, whether the compensatory of Pyk2 plays an important role in the regulation of angiogenesis and tumor growth should be further studied.

We have found that FAK could regulate the VEGFR2 expression in TNBC cells, and prior studies have also suggested that FAK could regulate AKT [17,19,20]. However, other studies have suggested that Sp1 is an important promoter of VEGFR2 and is regulated through AKT [50,51]. Therefore, it seems that VEGFR2 could be regulated through a FAK/AKT/Sp1 pathway. In addition, a recent study indicated that VEGFR2 could be regulated through programmed death ligand-1 (PD-L1) in ovarian cancer [52], and our recent study and another study also found that PD-L1 could be regulated by FAK [21,22], suggesting that VEGFR2 might be regulated through FAK/PD-L1 axis. However, the detail mechanism should still be investigated in future.

## 5. Conclusions

In summary, angiogenesis has been demonstrated to be a critical factor in tumor progression and metastasis. The strategies of antiangiogenic agents have benefitted hundreds of thousands of patients with cancer. However, clinical and preclinical observations indicate that these therapies may have limited efficacy owing to the complex gene regulation between tumor cells and their microenvironment. Our study showed that FAK is positively correlated with VEGFR2 and that FAK could regulate VEGFR2 and several angiogenic-related gene expressions in TNBC cells, suggesting that FAK has the potential to mediate tumor cell angiogenesis by regulating the VEGF/VEGFR2 feed-forward loop in TNBC cells. Therefore, targeting FAK could be another antiangiogenic strategy for TNBC treatment.

## Figures and Tables

**Table 1:** Association of FAK and VEGFR2 expressions in all breast cancer tissues, non-TNBC tissues and TNBC tissues.

**All Breast Cancer**	**VEGFR2, *n* (%)**		***p*** **-value**
	**Positive**	**Negative**	
High	15 (41.7)	8 (22.2)	
**FAK**			
Low	4 (11.1)	9 (25.0)	*p >* 0.05
**Non-TNBC**	**VEGFR2, *n* (%)**		***p*** **-value**
	**Positive**	**Negative**	
High	8 (34.8)	7 (30.4)	
**FAK**			
Low	3 (13.0)	5 (21.7)	*p >* 0.05
**TNBC**	**VEGFR2, *n* (%)**		***p*** **-value**
	**Positive**	**Negative**	
High	7 (53.8)	1 (7.7)	
**FAK**			
Low	1 (7.7)	4 (30.8)	*p <* 0.05

## Data Availability

The data presented in this study are available in the article.
